# Supplementary material for: High stress twinning in a compositionally complex steel of very high stacking fault energy
Source: Nat Commun. 2022 Jun 23;13:3598. doi: 10.1038/s41467-022-31315-2 (PMC9226120; doi:10.1038/s41467-022-31315-2)
Supplement: Supplementary file 1 — Supplementary Information [file 41467_2022_31315_MOESM1_ESM.pdf]

Supplementary Information for

High stress twinning in a compositionally complex steel of very high stacking fault energy

Zhangwei Wang[1*], Wenjun Lu[2*], Fengchao An[2], Min Song[1], Dirk Ponge[3], Dierk Raabe[3], Zhiming Li[1,3,4*]

1. State Key Laboratory of Powder Metallurgy, Central South University, 410083 Changsha, China.

2. Department of Mechanical and Energy Engineering, Southern University of Science and Technology, 518055, Shenzhen, China.

3. Max-Planck-Institut für Eisenforschung, Max-Planck-Str. 1, 40237 Düsseldorf, Germany.

4. School of Materials Science and Engineering, Central South University, 410083 Changsha, China.

* Correspondence to: z.wang@csu.edu.cn (ZW); luwj@sustech.edu.cn (WL); zhiming.li@csu.edu.cn (ZL).

This PDF file includes:

Supplementary Fig. 1 to 8

Supplementary Table 1 to 2

Supplementary References

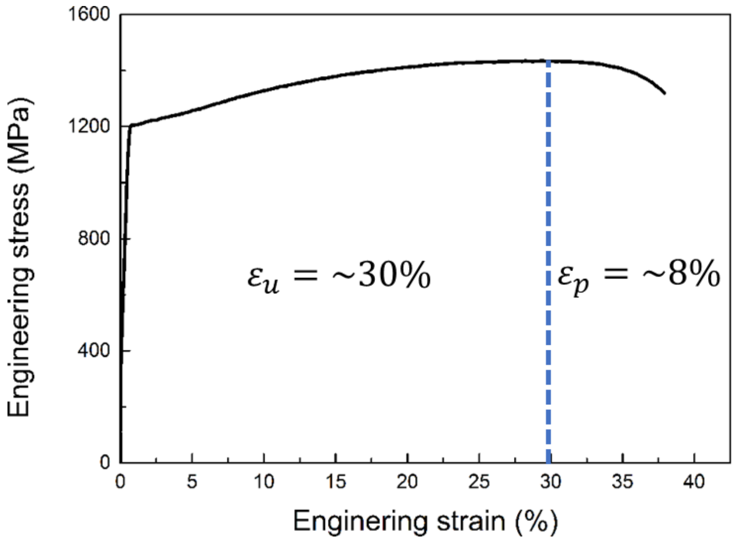

Supplementary Fig. 1 Engineering stress-strain curve of the CCS. Values of uniform elongation (ε_u) and post uniform elongation (ε_p) are marked in the figure.

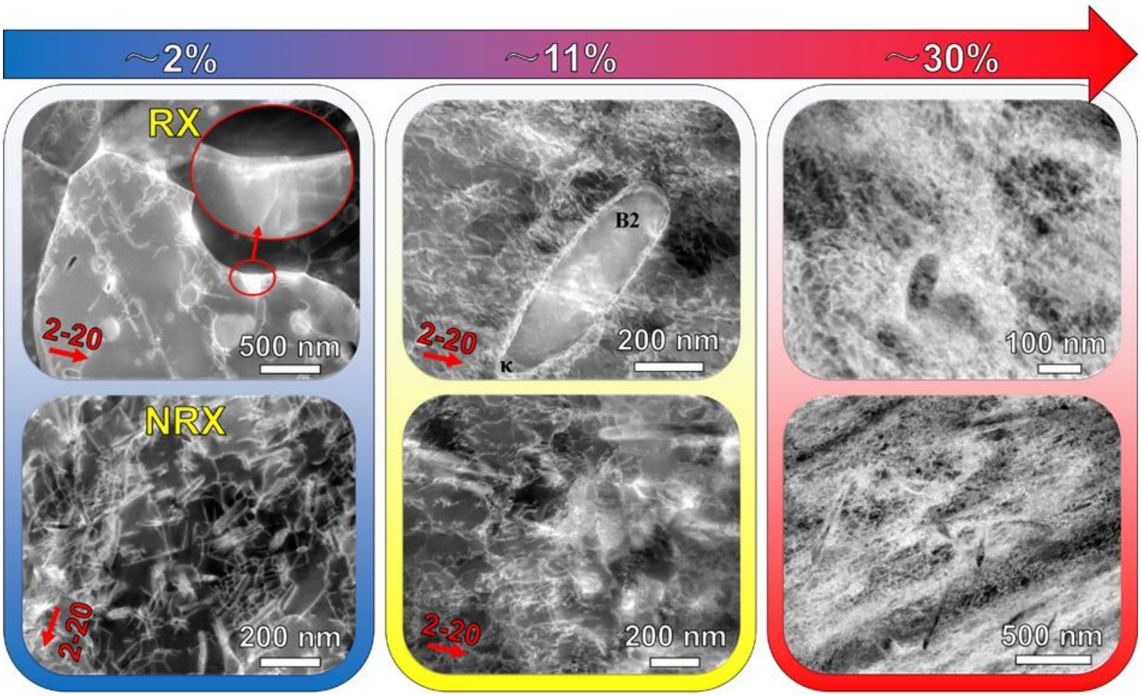

Supplementary Fig. 2 Deformation microstructures of the bulk CCS at different local strains (2%, 11%, and 30 %) based on LAADF-STEM observations. At an intermediate strain of 11%, the RX and NRX grains can be hardly differentiated anymore, as all grains show very high densities of dislocations (~3.12×10^{14} m^{-2}). The magnified image shows the slip of full dislocations at a strain of 2 %.

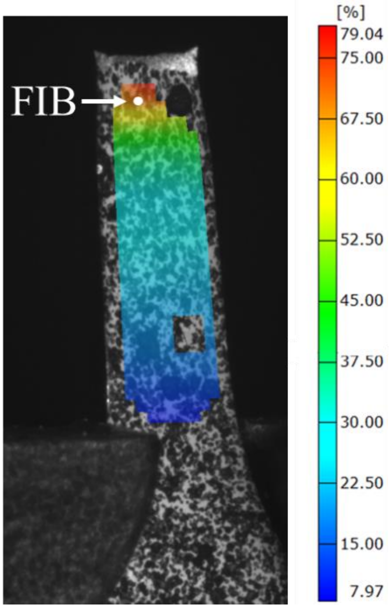

Supplementary Fig. 3 Digital image correlation (DIC) measurement of the local strain distribution of the tensile fractured sample. The region lifted out by FIB for deformation microstructure observations was at ~70 % local strain.

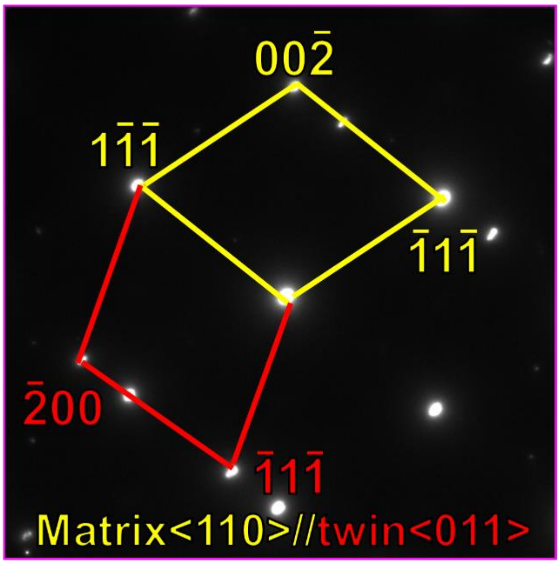

Supplementary Fig. 4 Index of SAED patterns in Fig. 3h. The twinning structure is clearly revealed.

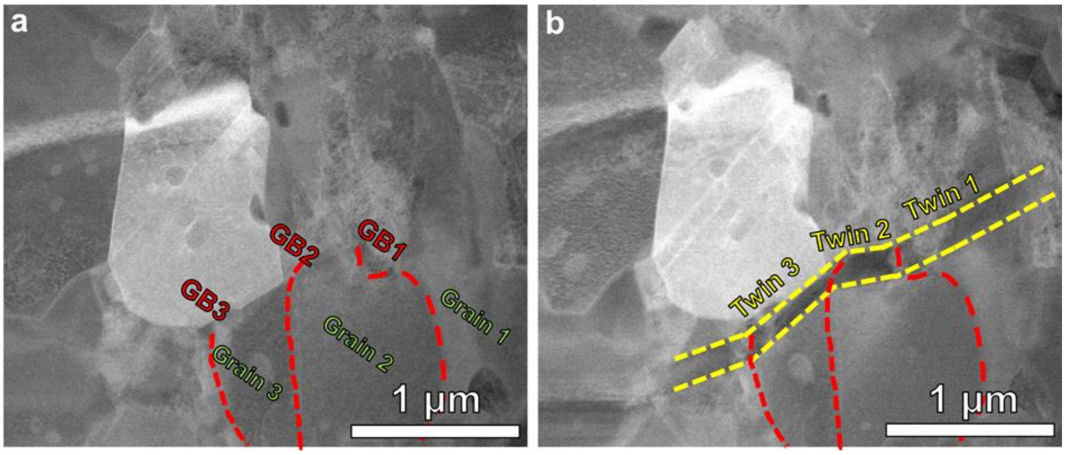

Supplementary Fig. 5 *In-situ* deformation via LAADF-STEM observations. a and b are magnified images corresponding to the images shown in Fig. 3f and 3g, respectively. The red dashed lines represent the grain boundaries. The yellow dashed lines outline the deformation twins formed during tensile loading. Twins pass through the grains and change directions depending on the misorientation angle between grains.

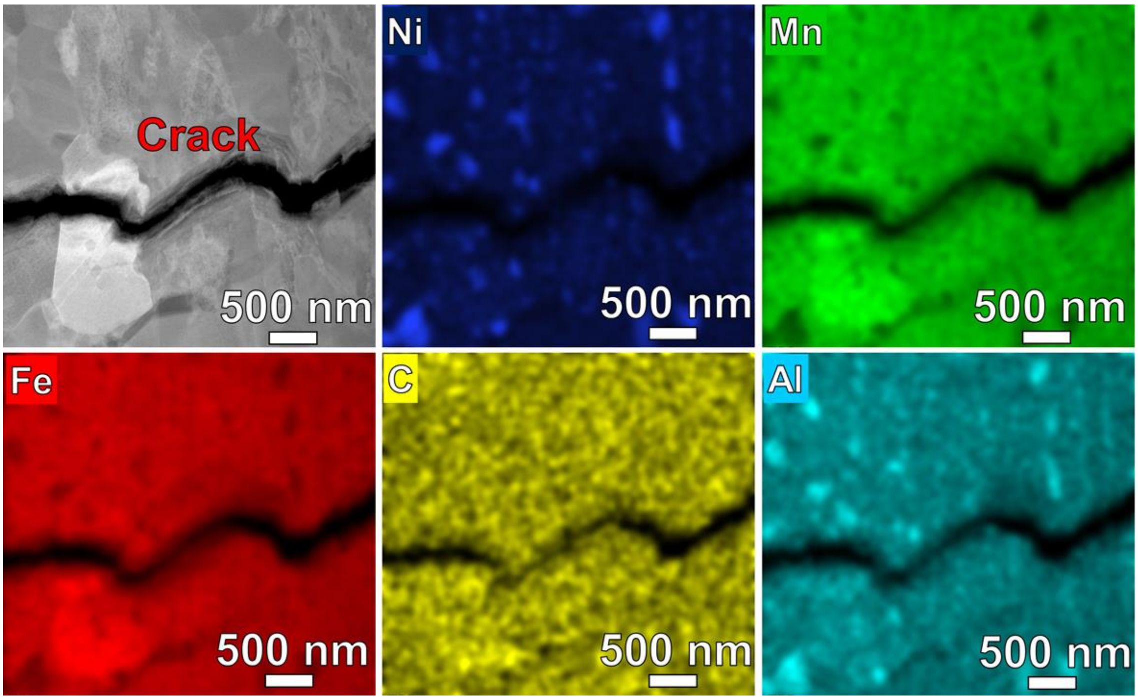

Supplementary Fig. 6 EDS maps showing the elemental distribution around the cracks after *in-situ* tensile test. The nucleation of crack was not observed at the incoherent interfaces between matrix and B2 particles. The crack went across different grains, showing an intragranular fracture mode.

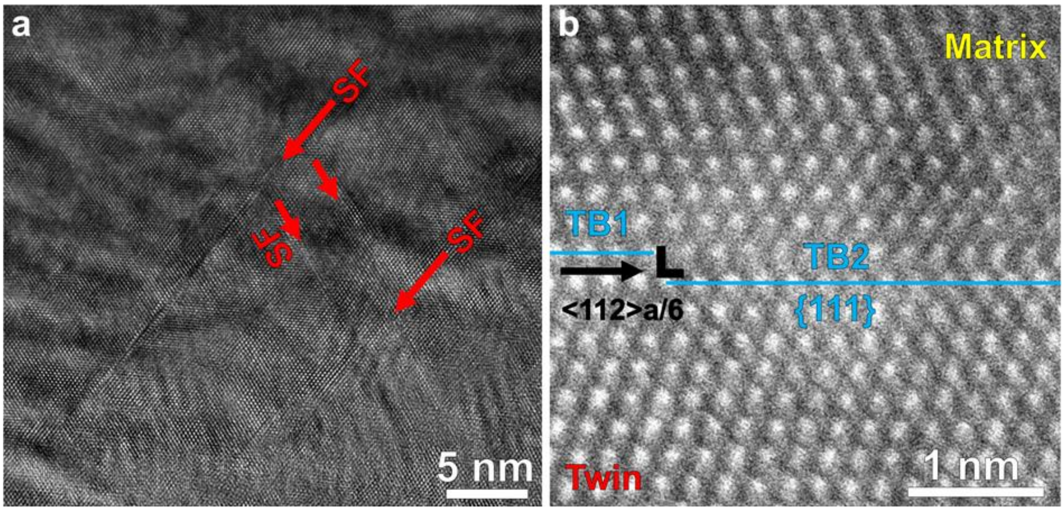

Supplementary Fig. 7 Formation of mechanical twins near the fracture area during the *in-situ* tests (imaged after the test). a, HRTEM reveals the formation of stacking faults (SFs) in the FCC matrix (marked by red arrows). b, HAADF-STEM imaging shows the formation mechanism of a mechanical twin in our CCS, which is associated with partial dislocation motion (leading partial with Burgers vector <112>a/6 indicated by a black arrow).

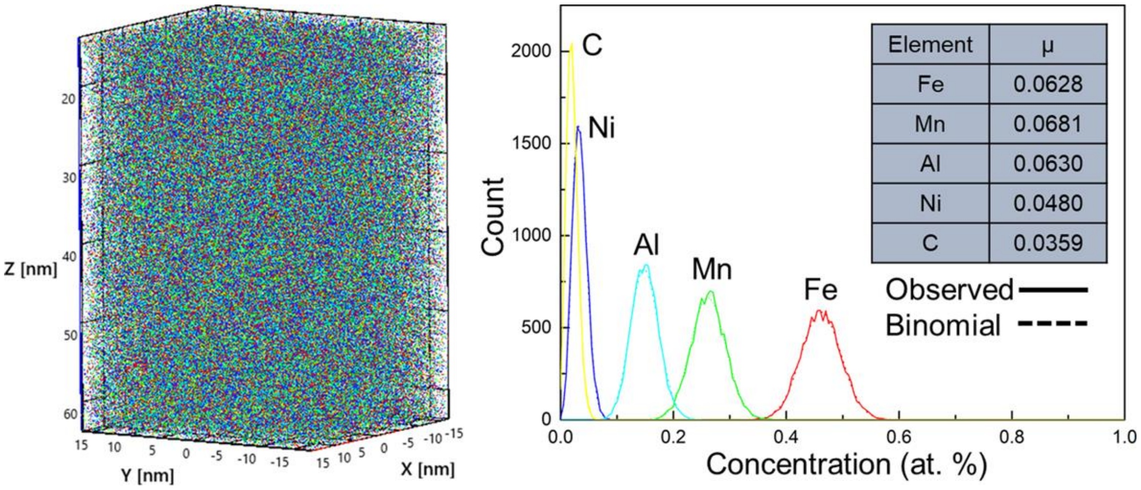

Supplementary Fig. 8 A typical APT map and frequency distribution analysis of the FCC matrix based on the chi-squared statistical test. The values of the normalized chi-squared parameter (μ) are very close to 0 for all five elements (Fe, Mn, Al, Ni, and C), which indicates the nearly ideal random distribution.

Supplementary Table 1 Values and functions used for the calculation of the SFE[1,2].

Parameter	Value and function
ρ(mol/m^2)	2.94×10^{-5}
σ(mJ/m^2)	9
$\Delta G_{Fe}^{\gamma\rightarrow\varepsilon}$(J/mol)	-2243.38 + 4.309 T
$\Delta G_{Mn}^{\gamma\rightarrow\varepsilon}$(J/mol)	-1000.00 + 1.123 T
$\Delta G_{Al}^{\gamma\rightarrow\varepsilon}$(J/mol)	2800 + 5 T
$\Delta G_{Ni}^{\gamma\rightarrow\varepsilon}$(J/mol)	1046 + 1.255 T
$\Delta G_{C}^{\gamma\rightarrow\varepsilon}$(J/mol)	-22166
$\Omega_{FeMn}^{\gamma\rightarrow\varepsilon}$(J/mol)	$2180+532(\chi_{Fe}-\chi_{Mn})$
$\Omega_{FeAl}^{\gamma\rightarrow\varepsilon}$(J/mol)	3339
$\Omega_{FeNi}^{\gamma\rightarrow\varepsilon}$(J/mol)	2095
$\Omega_{FeC}^{\gamma\rightarrow\varepsilon}$(J/mol)	42500
$\Omega_{MnC}^{\gamma\rightarrow\varepsilon}$(J/mol)	26910
$T_{Ne'el}^{\gamma}$(K)	$251.71+681\chi_{Mn}-1800\chi_{Ni}-1151\chi_{Al}-1740\chi_{C}$
$T_{Ne'el}^{\varepsilon}$(K)	$580\chi_{Mn}$
β^{γ}	$0.7\chi_{Fe}+0.62\chi_{Mn}+0.62\chi_{Ni}-0.64\chi_{Fe}\chi_{Mn}-4\chi_{C}$
β^{ε}	$0.62\chi_{Mn}-4\chi_{C}$

Supplementary Table 2 Comparison of calculated SFEs by our model with experimental results from the literature.

Compositions (wt. %)	Experimental methods	Experimental SFE (mJ/m^2)	Calculated SFE by current model (mJ/m^2)
Fe-18Mn-0.6C	TEM	13 ± 3 [3]	14
	XRD	17 ± 3 [4]	
	Neutron diffraction	21 ± 4 [5]	
	XRD	19 ± 3 [6]	
Fe-18Mn-1Al-0.6C	XRD	30 ± 2 [4]	20
Fe-18Mn-1.5Al-0.6C	TEM	30 ± 10 [3]	24
	Neutron diffraction	29 ± 3 [5]	
Fe-18Mn-2Al-0.6C	XRD	36 ± 2 [4]	27
Fe-18Mn-3Al-0.6C	Neutron diffraction	44 ± 5 [5]	33

Supplementary References:

1 Curtze, S., Kuokkala, V. T., Oikari, A., Talonen, J. & Hänninen, H. Thermodynamic modeling of the stacking fault energy of austenitic steels. *Acta Mater.* **59**, 1068-1076 (2011).

2 Yoo, J. D. & Park, K.-T. Microband-induced plasticity in a high Mn–Al–C light steel. *Mater. Sci. Eng. A* **496**, 417-424 (2008).

3 Kim, J., Lee, S.-J. & De Cooman, B. C. Effect of Al on the stacking fault energy of Fe–18Mn–0.6C twinning-induced plasticity. *Scr. Mater.* **65**, 363-366 (2011).

4 Jin, J. E. & Lee, Y. K. Effects of Al on microstructure and tensile properties of C-bearing high Mn TWIP steel. *Acta Mater.* **60**, 1680-1688 (2012).

5 Jeong, J. S., Woo, W., Oh, K. H., Kwon, S. K. & Koo, Y. M. In situ neutron diffraction study of the microstructure and tensile deformation behavior in Al-added high manganese austenitic steels. *Acta Mater.* **60**, 2290-2299 (2012).

6 Jeong, K., Jin, J.-E., Jung, Y.-S., Kang, S. & Lee, Y.-K. The effects of Si on the mechanical twinning and strain hardening of Fe–18Mn–0.6C twinning-induced plasticity steel. *Acta Mater.* **61**, 3399-3410 (2013).
