# Supplementary material for: High stress twinning in a compositionally complex steel of very high stacking fault energy
Source: Nat Commun. 2022 Jun 23;13:3598. doi: 10.1038/s41467-022-31315-2 (PMC9226120; doi:10.1038/s41467-022-31315-2)
Supplement: Supplementary file 3 — Description of Additional Supplementary Files [file 41467_2022_31315_MOESM3_ESM.pdf]

Description of Additional Supplementary Files

File Name: Supplementary Movie 1
Description: In-situ TEM observation of deformation microstructure evolution in the CCS under tension. The scale bar in the video is 1 μm.
